# Impacts of Self-Regulated Strategy Development-Based Revision Instruction on English-as-a-Foreign-Language Students' Self-Efficacy for Text Revision: A Mixed-Methods Study

**DOI:** 10.3389/fpsyg.2021.670100

**Published:** 2021-07-15

**Authors:** Jing Chen, Lawrence Jun Zhang, Xiao Wang, Tingting Zhang

**Affiliations:** ^1^College of Foreign Languages, Huazhong Agricultural University, Wuhan, China; ^2^Faculty of Education and Social Work, University of Auckland, Auckland, New Zealand; ^3^Teaching and Learning Group, The Royal New Zealand Police College, Wellington, New Zealand

**Keywords:** self-regulated strategy development, self-efficacy, text revision, EFL writing, classroom intervention

## Abstract

This mixed-methods study investigated the impacts of the self-regulated strategy development (SRSD) model on the self-efficacy of students for text revision in English-as-a-foreign-language (EFL) writing at the tertiary level. An SRSD treatment group and a comparison group were involved in this quasi-experimental design research. Both groups completed a self-efficacy scale before and after the instruction, and six SRSD-trained students participated in pre- and post-test interviews. The quantitative analyses did not detect any significant differences between groups, suggesting that the SRSD instruction did not influence the self-efficacy of participants for text revision. The qualitative findings provided insights into the quantitative results. The interview data indicated that the interviewees might have overestimated their revision abilities before instruction and, with the relatively more accurate estimation of their abilities resulting from receiving the SRSD instruction, the over-time comparison of their responses to the self-efficacy scale did not reveal any statistically significant changes. Our findings suggest that students might have recorded evidence of closer calibration between judgments of their revision abilities and their actual performance after SRSD instruction. The implications of the findings were discussed and directions for further studies were provided.

## Introduction

Revision is a critical part of the writing process as well as writing instruction not only in one's first language (L1) but also in a foreign language (FL). It is of great instructional significance as inexperienced L1 and FL writers need to learn how to revise effectively to become proficient writers. It also provides an opportunity for instructors to teach students about the characteristics of effective writing in a way that will carry over to future writing (MacArthur, 2015, 2018). Revision, however, is unanimously depicted as a complex activity in the theoretical models of revision, as reported in many studies in the field of psychology and educational psychology, which are either specifically conceptualized for the revising process (e.g., Butterfield et al., 1996; Hayes, 1996) or being embedded in the more general writing architecture (e.g., Hayes and Flower, 1980). It is not only a cognitively complex task that requires coordination and management of various kinds of knowledge and skills (e.g., Butterfield et al., 1996; Kellogg, 1996; Chanquoy, 2009; MacArthur, 2015, 2018; Zhang et al., 2018; Li and Zhang, 2021), but also a socially mediated meaning-making activity that takes account of the implied reader, text, and thought (e.g., Chanquoy, 2001; Myhill and Jones, 2007). Hence, novice or inexperienced L1 and FL writers may find it difficult or uncomfortable to execute (Zhang and Cheng, 2020). Moreover, most writing tasks in the school context lack authentic audiences or communicative purposes, which seldom motivates developing writers “to engage in the extra effort needed to revise” (MacArthur, 2015, p. 274). Thus, it is also necessary to take affective and confidence matters of students into account when researching revision, since the challenges students face in writing might as much be related to their affective factors as to cognitive factors (MacArthur, 2015, 2018).

It is postulated that individuals who have more confidence in performing successfully in a given domain are more likely to “expend effort and persist at difficult tasks” in that area (Schunk et al., 2014, p. 6). Beliefs of one's perceived capabilities to “organize and execute the courses of action required to attain designated types of performance,” which is a significant construct of motivation, have been postulated by Bandura as self-efficacy (Bandura, 1986, p. 391). Self-efficacy, which is rooted in the fundamental belief that one has the power to effect changes by one's actions (Bandura, 2004; Klassen and Usher, 2010), can guide people's options, efforts, and level of perseverance with tasks (Paris and Winograd, 1990; Pintrich and Schunk, 2002; Bong, 2006; Zhang and Zhang, 2019; Harris and Leeming, 2021). Given the complexity of effective revision and the lack of ideal motivational conditions in school contexts, self-efficacy becomes particularly critical in the domain of revision.

Informed by social cognitive theory, the employment of self-regulated learning strategies allows students to effectively monitor and control their learning progress, thus potentially contributing to their self-efficacy (Zimmerman, 2000; Schunk and Zimmerman, 2007; Lantolf and Poehner, 2008; Harris et al., 2010). The relationship between self-regulation and self-efficacy in L1 writing has been well-documented (e.g., Fidalgo et al., 2008; MacArthur and Philippakos, 2010; Bruning and Kauffman, 2016; Paul et al., 2021). In the field of EFL/ESL writing, empirical evidence in support of the positive influences of self-regulated strategy use on self-efficacy of students has been obtained (e.g., Zhang et al., 2016; Bai and Guo, 2018; Chen and Zhang, 2019; Teng and Zhang, 2020). This study thus leveraged on the self-regulated strategy development (SRSD) model, which includes explicit teaching of strategies for self-regulating strategy use and writing behavior, to improve students' self-efficacy in the domain of text revision.

Moreover, the SRSD model is designed to directly improve students' self-efficacy and motivation. Instructional procedures for fostering self-efficacy, including goal setting, self-monitoring, self-instruction, and self-reinforcement, are included in the model (Graham et al.'s, 2005; Harris et al., 2015; Harris and Graham, 2016). Previous SRSD studies in L1 contexts have found positive effects on students' writing self-efficacy (Graham et al.'s, 2005; MacArthur et al., 2015). However, relatively few studies have been conducted in EFL contexts, and little attention has been paid to the specific domain of revision in EFL writing given the challenges of conducting intervention studies (Huang and Zhang, 2020; Li et al., 2020). This study aimed to address the research gaps by exploring the possible changes in self-efficacy of EFL students for text revision resulting from receiving the SRSD instruction.

## Literature Review

### SRSD for Text Revision in Writing

The SRSD model, as used in L1 English contexts, in the form of interactive, scaffolded, and explicit instruction, aims to facilitate students' development of strategies and knowledge for accomplishing targeted writing tasks and self-regulation procedures needed to monitor and manage their writing process (Harris and Graham, 2016). Motivation and attitudes are targeted along with knowledge and strategies, as one of the major goals of the SRSD approach is to help learners develop positive attitudes toward writing and view themselves as writers (Harris et al., 2015). Hence, attributions to effort and strategy deployment are explicitly developed in the SRSD model to improve the self-efficacy of students (Harris et al., 2011). The importance of student effort in learning is emphasized and rewarded throughout the instructional program to facilitate the development of positive attitudes (Harris et al., 2015). Although this model was originally developed to help students with learning disabilities, it has been successfully implemented with various types of students, with or without learning disabilities (Graham et al., 2013). Six instructional stages, namely, developing background knowledge, discussing, modeling, memorizing, supporting, and independent performance, are implemented in the SRSD model. Throughout the instructional process, teachers model the writing process aloud, with input and support from students, and demonstrate explicitly how to use writing strategies as well as self-regulatory strategies. Previous research showed that observing models that explain and demonstrate strategies could foster learners' self-efficacy for learning and boost their motivation to learn (Schunk and Zimmerman, 2007; Schunk and Mullen, 2018).

In L1 contexts, the application of SRSD to teach revision strategies has produced positive effects on participants' revising behavior and writing quality (e.g., Schnee, 2010; Song and Ferretti, 2013). For instance, Song and Ferretti (2013) explored the effectiveness of SRSD revising instruction that targeted the use of argumentation schemes and critical questions for college students. Results showed that the revision strategy instruction had positively impacted the revision of participants. They thus concluded that teaching revising strategies using the SRSD is “a promising method” for improving argumentative essays of college students (p. 88).

The SRSD model was also utilized to teach EFL/ESL students how to revise effectively (e.g., De La Paz and Sherman, 2013). De La Paz and Sherman's (2013) investigation showed that participants made more meaningful changes, revised longer text segments, and produced more revisions that improved text after learning the revision strategy. These results provided evidence for the efficacy of the SRSD instruction in the context of revision with students who are English learners.

In sum, researchers have modified the SRSD model and applied it in teaching revision strategies in both L1 (e.g., Schnee, 2010) and ESL/EFL writing classrooms (e.g., De La Paz and Sherman, 2013), and improvements were shown in revision performance and writing quality of students. Nevertheless, few studies have explored the instructional effects on the self-efficacy of students for revision as it was beyond their research scope. The following sections review the operationalization of self-efficacy in the domain of text revision and studies indicating writing self-efficacy changes resulting from self-regulation strategy-based writing instruction.

### Self-Efficacy for Text Revision in Writing

The complexity of revision as depicted by existing cognitive models of revision processes (e.g., Flower and Hayes, 1981; Flower et al., 1986; Hayes et al., 1987; Hayes, 1996) and lack of ideal motivational conditions in many school contexts (MacArthur, 2018) render self-efficacy necessary in the domain of revision in L1 contexts. To accurately investigate the perceived capabilities of students for revision, which is a specialized writing activity that makes use of all the processes in writing including proposing, translating, and reading (Hayes, 2012), this study drew on previous research that operationalized self-efficacy for revision as a specific domain. Informed by Bruning et al. (2013), self-efficacy scales for writing in general tended to “broadly sample writing-related skills and tasks” and were not ideal for eliciting information about specific dimensions of writing, such as revision (Bruning et al., 2013, p. 36).

Despite the bulk of studies on self-efficacy for writing in general, research in self-efficacy for revision as a specific domain is limited. One self-efficacy study that targeted the domain of revision is Zimmerman and Kitsantas's (2002) investigation in an L1 writing context. To examine the instructional effects of modeling and social feedback on college students' revision skills and their self-regulatory perceptions, including self-efficacy, they operationalized self-efficacy for revision as students' perceived abilities to solve specific revision problems (e.g., combining simple kernel sentences into non-repetitive ones), which were taught in their teaching intervention. As their construct of self-efficacy for revision was specific to their experimental purposes, it provides little information on the perceived capabilities of students in engaging in revision as a general domain.

In a more recent study in this line, Chen and Zhang (2019) proposed a two-factor of self-efficacy for text revision in EFL writing: self-efficacy for high-level text revision (i.e., students' judgments of their abilities to revise high-level text features such as style and organization) and self-efficacy for low-level text revision (i.e., students' judgments of their abilities to perform revisions regarding low-level text features such as local, surface mechanical, grammatical, and lexical problems). The two focal factors are consonant with the two main types of revision processes described in cognitive models of revision (e.g., Hayes, 1996, 2004, 2012). An intentional, reflective process involving a systematic evaluation of high-level text features combined with detection and resolution of problems regarding lower-level text features was reported. Chen and Zhang (2019) scrutinized the psychometric properties of this two-factor model with responses from 756 EFL college students. Their results of factor analyses confirmed the proposed two-factor model.

To sum up, limited attention has been paid to the exploration of self-efficacy in the specific domain of revision in writing, particularly the value of teaching self-regulation strategies in affecting self-efficacy for revision in EFL contexts. Pedagogically, it is of great significance to investigate if students could become more confident in engaging in revision, a cognitively demanding yet crucial task for writing, after being equipped with self-regulation strategies.

### Relationships Between SRL Strategies and Self-Efficacy in EFL Writing

Self-efficacy is of great educational significance as it can influence learners' academic behaviors, such as the level of perseverance with tasks (e.g., Woodrow, 2011) and the degree to which learners engage in metacognitive monitoring (e.g., Moos, 2014; Zhang and Zhang, 2018). More importantly, self-efficacy could influence students' utilization of SRL strategies in learning (Bandura, 1986, 1997). In the field of writing, many empirical studies have reported the statistically significant, positive correlations between SRL strategies and students' writing self-efficacy in L1 contexts (e.g., Bruning et al., 2013; Limpo et al., 2014; MacArthur et al., 2015). For instance, MacArthur et al.'s study 2015 with college students showed that after receiving the SRSD writing instruction, the students reported significantly higher self-efficacy for writing tasks and processes on a self-efficacy scale than the comparison group. In their study, self-regulated strategies, including goal setting, task management, progress monitoring, and reflection, were taught. These authors attributed gains in participants' self-efficacy to mastery experience gained through the SRSD instruction.

Consistent with these findings obtained in L1 settings, studies in EFL/ESL writing have also identified the close link between writing self-efficacy and self-regulation strategy employment (e.g., Bai and Guo, 2018; Sun and Wang, 2020; Teng and Zhang, 2020), as is summarized about the role language learning strategies in improving learner performance (Zhang et al., 2019). For example, Teng and Zhang (2020) leveraged the SRSD model to teach EFL college students in academic writing. After the 5 month SRL strategy-based writing instruction, participants reported increased levels of writing self-efficacy as measured by a writing self-efficacy scale, particularly their performance self-efficacy (i.e., perceived abilities to accomplish writing tasks) and linguistic self-efficacy (i.e., perceived abilities to utilize knowledge in text generation). The results provided evidence in support of the proposition that successful use of self-regulation strategies could contribute to strengthened writing self-efficacy in ESL/EFL contexts.

Despite the evidence indicating the positive relationship between self-regulation strategies and writing self-efficacy, other studies suggested otherwise (e.g., Graham et al.'s, 2005; García-Sanchez and Fidalgo-Redondo, 2006; Mills, 2012). For instance, in Graham, Harris and Mason (2005) study in an L1 context, students' writing self-efficacy was not influenced in the SRSD conditions as indicated by their responses to the items on a self-efficacy scale before and after instruction. The authors attributed the non-significant relationship to the inaccuracies in young writers' judgments of their abilities.

Although preliminary evidence in support of the relationship between these two variables was reported in EFL writing contexts, in the domain of revision, it is unclear whether the model applied to the process of revision is still valid or not. Given the inconsistency in the motivational effects of SRSD in L1 writing as reported in the former investigations, as well as the relatively limited research exploring this issue in L2 contexts, especially in the domain of revision in EFL writing, more studies are needed to investigate the effectiveness of influencing self-efficacy of students for revision *via* targeting their use of SRL strategies in the revising process.

## Method

### Research Design

The current study was designed to address the research gaps mentioned above. This study is part of a larger research project, which adopted the SRSD model to teach college students revision strategies in EFL writing and examined its effects on their metacognitive knowledge, self-efficacy for text revision, revision performance, and written text quality using a quasi-experimental design. The current study focused on the instructional effects of the SRSD revision instruction on students' self-efficacy for text revision. Two intact classes of students participated in this study with one class receiving the SRSD instruction and one the regular writing instruction required by the University. Details are provided in the ensuing sections.

A convergent parallel mixed-methods design was utilized to obtain participant views within the context of a quasi-experimental treatment (Creswell and Creswell, 2018). This study drew on the pragmatic worldview on research as we believe that diverse types of data could provide a more holistic understanding of self-efficacy than quantitative or qualitative data alone (Cresswell, 2014; Creswell and Creswell, 2018). Besides the quantitative information on the trends of self-efficacy of students at different time points yielded from using a self-efficacy scale, semi-structured interviews were utilized to depict a fuller portrait of the complex issue of self-efficacy. The overall objective of this research is to investigate the possible changes in self-efficacy of students in the specific domain of revision in EFL writing after receiving the SRSD treatment. Two research questions guided this study:

1. Do SRSD trained students have higher ratings for the self-efficacy scale compared to untrained students after instruction?
2. How does the SRSD revision instruction influence self-efficacy of students for text revision as reported in the interviews?

### Participants

Convenience sampling was employed in the recruitment of student participants for easy accessibility. Two intact classes participated in this project, and they were assigned into one of the two instructional conditions. All participants were second-year undergraduates with an average age of 18.97 (*SD* = 0.72). Independent samples *t*-tests were performed on the baseline data of the two groups, and no statistically significant differences were found between the groups. The two groups were comparable in terms of their age, length of formal English learning, and gender proportion (see Table 1). The teacher volunteers were experienced EFL teachers and were of similar educational backgrounds. As they both had limited experience with SRSD instruction, they were randomly assigned to either group.

**Table 1:** Participant's demographic information.

**Group**	***n***	**Major**	**Male%**	**Age**	**Length of English learning (years)**
Treatment group	28	Education	46.4	18.94	9.65
Control group	28	Translation	50.0	18.88	9.88

### Measures

#### The Second Language Text Revision Self-Efficacy Scale (L2TRSS)

The Second Language Text Revision Self-Efficacy Scale (L2TRSS), a 17-item scale developed to evaluate the perceived capabilities of students to perform activities involved in text revision in EFL writing (Chen and Zhang, 2019), was applied in this study. The L2TRSS measured two dimensions of self-efficacy for text revision: self-efficacy for high-level text revision and self-efficacy for low-level text revision. This two-factor structure of self-efficacy for text revision is informed by cognitive models describing two main types of processes involved in text revision: a more or less automatic process involving detection and modification of low-level text problems along with a more intentional, systematic process of evaluation and refinement of high-level text features (Hayes, 1996, 2004). The two factors are in line with revision outcome models that categorize external revisions into low-level, surface revisions or higher-level, deep revisions (e.g., Faigley and Witte, 1981; Lindgren and Sullivan, 2006; Chanquoy, 2009).

The L2TRSS evaluates EFL learners' judgments of their abilities to perform revision activities concerning high-level text features (e.g., meaning or organization-related revisions) and their perceived capability to revise at the low level (e.g., surface, local text elements). Factor analyses were performed in their study to assess the psychometric properties of the scale, and their results showed that the reliability (e.g., Cronbach's alpha coefficient of internal reliability for both subscales was over.85) and validity (e.g., factor analyses results: *x*^2^ = 472, *df* = 116, *CFI* = 0.925, *TLI* = 0.912, *RMSEA* = 0.101, [0.091,0.110], *SRMR* = 0.05) of it were satisfactory (Chen and Zhang, 2019). In their study, self-efficacy scores of students had a statistically significant, positive correlation with their writing scores (*p* < 0.01). The researchers used a 10-point Likert scale with gradations from 0 (*I cannot do it*) to 10 (*Highly certain that I can do*) instead of a 0–100 response format that was advocated in many self-efficacy studies (e.g., Pajares et al., 2001) mainly because respondents in their study expressed confusion in using a scale of that specificity and only used tenths. As the validated research setting is highly similar to that of the current research, namely, in which students learnt EFL writing at the tertiary level, we adopted the 0–10 response format proposed in their study. In the current study, the internal reliability of the two factors were 0.892 and 0.921, much higher than the benchmark value of 0.70. Appendix A displays the factors and items of the L2TRSS.

#### Semi-structured Interviews

Semi-structured interviews, which serve a middle position between structured and unstructured interviews, guide interviewees to focus on a target topic area with a set of pre-prepared questions or prompts while allowing for follow-up developments and elaboration *via* their open-ended format (Dörnyei, 2007). The same guiding questions make answers comparable across different respondents to a certain degree, as the “-structured” part in the name indicates; this type of interview also makes room for “variation or spontaneity in responses,” hence the “semi-” part (Dörnyei, 2007, p. 136). Moreover, they are helpful for researchers to collect in-depth information concerning people's opinions, ideas, and experiences (Fraenkel et al., 2012). Hence, they were employed in this study for their usefulness in adding depth and richness to information elicited from the self-efficacy scale that employs closed questions (Dörnyei, 2007). The pretest interviews aimed to explore the previous revision experience of participants and their perceived revising ability, and the posttest interviews focused on their current understanding and belief. Participants were also encouraged to specify any perceived changes in their understanding of revision, their revision behaviors, and their confidence in revision capacity, for instance, knowledge regarding the nature and purpose of revision they were not aware of or revision strategies that they did not use prior to the treatment.

### Procedures for SRSD Instruction

**Figure 1:**
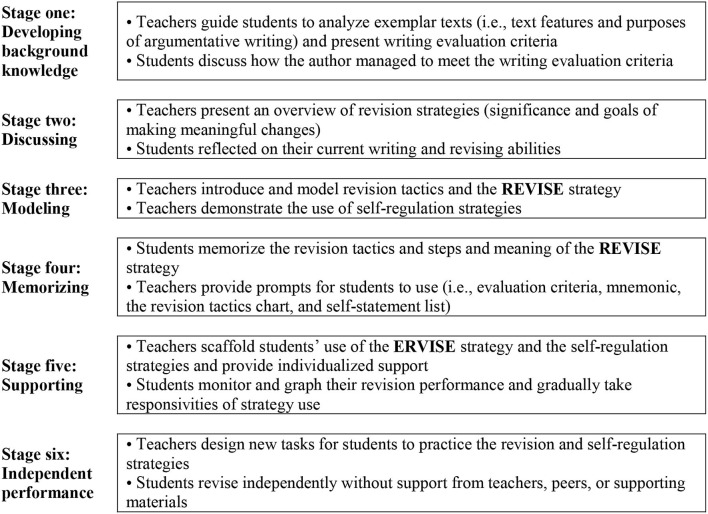
Six stages of the SRSD revising instruction (adapted from Harris et al., 2015).

The SRSD revision instruction in this study was designed following the core principles of the SRSD model (Graham et al., 2012, 2013). The revision strategy REVISE (Read the essay; Evaluate the problems; Verbalize how to fix the problems; Implement the changes; Self-check; End by rereading) was taught (see Harris, 2008, for detailed explanations). The six recursive stages of the SRSD revision instruction are depicted in Figure 1. The description of the instructional process presented below focuses on how motivation and self-efficacy were addressed during instruction.

The instruction began with teachers developing students' background knowledge of argumentative writing and writing evaluation criteria. During this stage, teachers also helped students to identify whether their writing and revising performance were hindered by negative self-statements (e.g., *I'm not good at it*) and showed them how to utilize positive self-statements (e.g., *I can do this if take my time*). Then teachers introduced the strategies to be learned, along with the instructional goals and the significance of revision. Teachers also asked questions to elicit the reflection of students on their revision process to explore further their current revision ability. This was carried out in a positive, collaborative manner with an emphasis on the fact that they have not mastered the target strategies. The importance of students' efforts was emphasized to facilitate the development of motivation and positive attributions. In the modeling stage, teachers modeled how and when to use the REVISE strategy and revision tactics (i.e., adding, moving, deleting, and rewriting) (Fitzgerald and Markham, 1987). They also demonstrated self-regulation strategies, including self-instructions (e.g., *I'd better rewrite this reason as it doesn't seem to support my idea*), self-evaluation (e.g., *Is the reason convincing enough?*), and self-reinforcement (e.g., *I think the revised part is much better!*). Students participated in engaging activities designed to help them recite the revision tactics and the steps and the meaning of the REVISE strategy. They were encouraged to monitor their use of the revision strategies and graph their revision performance by counting the number of revisions.

The improvements in their revision performance could contribute to their motivation for revising. Additionally, individualized support was provided to students who were confronted with difficulties in revising and writing. For instance, to help a student who constantly wrote very short essays, this student and the teacher set a personal goal for his performance: adding at least one reason to support his position and an example to support each reason. He was encouraged to self-reinforce his success by counting the number of argument elements in his essay. Teachers gradually removed their support until students could independently use the strategies.

### Treatment Fidelity

The SRSD instructors participated in 3 weekly training workshops which introduced the stages and features of the SRSD model and the main classroom activities involved. They were provided with all the necessary materials, including lesson plans, checklists, PPT slides, writing prompts, and answer sheets. The instructor's role as a mediator was emphasized, and they were reminded that they need to activate the background knowledge of students, stimulate their interest to learn new strategies, and guide them to collaborate with peers. The instructors demonstrated a demo lesson and engaged in critical reflection about their teaching practices afterward. They also anticipated the possible difficulties they might be confronted with during the instruction and proposed solutions accordingly.

The fidelity of treatment implementation was evaluated using a checklist of lesson components and ratings of the key elements of the SRSD model. The key components were rated on a 3-point scale (“0” = element absent, “1” = element implemented with modification, “2” = element implemented as designed). One of the researchers observed and completed the checklist for all SRSD lessons and an EFL teacher evaluated a random 20 percent of the audio recordings of the lessons for each SRSD instructor. Overall, the SRSD instruction was implemented as in lesson plans with high fidelity in the treatment group. Across the two instructors and all lesson components, the average rating was 1.82 (*SD* = 0.16).

### Data Collection

All groups completed the L2TRSS before and after the instruction. Six volunteers from the treatment condition participated in the pre- and post-interviews. All interviews were conducted individually in a quiet setting; each interview lasted for 30 to 45 min. Appendix B displays the pre- and post-test interview schemes.

After the participating teacher completed the training workshops (an hour each time), the 8 week SRSD writing instruction began (once a week for 90 min). The comparison group received regular writing instruction required by the University curriculum and syllabus. All groups practiced using the same writing tasks in their writing textbook and received the same length of writing instruction. To ensure participants in the comparison group were not disadvantaged, they were offered the SRSD instruction after the research and were provided with all the resources used in the SRSD groups.

### Data Analysis

#### The Second Language Text Revision Self-Efficacy Scale (L2TRSS)

Points chosen by participants on the 10-point scale were coded as scores for the items; the composite score was used as an indicator of the overall level of participants' self-efficacy of their ability to revise. To examine if the self-efficacy scores of the two groups develop differently over time, two-way mixed ANOVA was employed with time (pretest and posttest) and instructional conditions (treatment and comparison) as the independent variables and self-efficacy scores as the dependent variable. Prior to the test, the assumption of normality was assessed by the Shapiro–Wilk test of normality (*p* > 0.05) along with P-P plots, and the results indicated that the data were approximately normally distributed. The assumption of homogeneity of variances and covariances, as measured by Levene's test (*p* > 0.05) and Box'es M test (*p* > 0.05), was also met (Field, 2013).

#### Semi-structured Interviews

Interview data were analyzed qualitatively in terms of understanding of participants of their perceived ability in carrying out revision activities. The audio-recordings, collected over six sessions of interviews, were transcribed, and the transcripts were presented to the interviewees, who were invited to change or delete statements that were inconsistent with their intended meaning. The revised transcripts were translated into English; the translated version was verified for accuracy by two high-proficient bilinguals, with one translating the transcripts from English to Chinese and the other translating them back from Chinese to English. One researcher coded the whole data, and another researcher coded a random 25 percent. All transcripts were imported to the program NVivo 12, which greatly facilitated checking and cross-checking of the consistency of coding. The inter-coder reliability was acceptable (i.e., the percentage of agreement between the two codes was over 80%). In cases where the two coders find the concept conveyed by text chunks could not be neatly categorized, the third researcher coded these parts and disagreements were resolved *via* discussion.

Data coding started with us familiarizing ourselves with the transcripts to reflect on their overall meaning and the tone of the ideas (Cresswell, 2014). In reading, we made marginal annotations on “noticing” and highlighted texts that required further analytical reflection, then moved on to more “detailed and systematic engagement” with the data (Braun et al., 2018, p. 11), involving bracketing chunks of text and identifying underlying meanings succinctly throughout the dataset. The chunks that conveyed similar meaning were clustered together into a broader group with a label, or a code attached; this code was abbreviated from the underlying meaning and represented experiences, ideas, attitudes, or feelings identified in the data at a more abstract level (Ellis and Barkuizen, 2005). In this way, the data were reduced into collated chunks of text and were organized around meaning-patterns. With the help of the program NVivo 12, a set of “tree nodes” was produced after line-by-line scrutinizing and coding.

Having identified these initial codes, we looked for relationships between them to organize codes into higher-level categories; these categories, or coherent clusters of meaning, helped to illustrate “a particular aspect of the dataset” (Braun et al., 2018). Codes were collated into themes in light of the interrelationships or similarities between them. This analysis was conducted both horizontally for common themes that surfaced from all participants' responses to interviews conducted at the same point in time (i.e., pretest interviews), as well as vertically for over-time developments within each respondent's data. In doing so, some codes, which were initially clustered together, were separated as substantial data emerged to support the existence of those codes, as independent entities.

Besides analyzing bottom-up, we also approached the transcripts in a top-down manner using the relevant theories and frameworks that underpin this study and potential codes were generated based on them. The data were explored and tagged with the predetermined codes. We also reflected on the data and the emergent themes to determine if there was more evidence that could be associated with them and moved between the transcripts and codes and themes recursively. The dual approach of inductive, bottom-up and deductive, top-down for data analysis iteratively allowed for the discovery of disconfirming data and the decision to delete themes that could not “tell a coherent, insightful story” about the data regarding the research questions (Braun et al., 2018, p. 12).

## Results

### Scores on the L2TRSS

Descriptive information on the ratings for the subscales of L2TRSS is presented in Table 2. No significant difference was found between the two groups in their pretest ratings. Two-way mixed ANOVA showed that there was no statistically significant interaction between the instructional condition and time on scores of *self-efficacy for high-level text revision* [*F*_(1,54)_ = 0.32, *p* = 0.57, partial η^2^ = 0.01], suggesting the two groups developed similarly in their ratings for *self-efficacy for high-level text revision* over time. There was also no significant interaction in mean ratings for *self-efficacy for low-level text revision* [*F*_(1,54)_ = 0.02, *p* = 0.90, partial η^2^ = 0.00], indicating minimal variance in the ratings for this subscale at different time points.

**Table 2:** Descriptive information on L2TRSS scores for each group.

		**Pre-test**			**Post-test**		
**L2TRSS subscale**	**Group**	***N***	***M***	***SD***	***N***	***M***	***SD***
Self-efficacy for high-level text revision	Treatment group	28	5.55	1.12	28	5.76	1.19
	Control group	28	6.04	0.97	28	6.45	1.00
Self-efficacy for low-level text revision	Treatment group	28	6.35	1.31	28	6.77	1.09
	Control group	28	7.02	1.37	28	7.38	1.24

### Interview Data

#### Pretest Interview

A considerable number of comments from the pretest interviews suggested that they lacked the motivation to revise while being overconfident in their revision abilities. As can be seen from Table 3, two themes with two codes subsumed under each emerged in qualitative analysis; one theme was pertinent to the contexts in which revision would not be attempted or continued, and the other was concerned with their overestimation of their revision capabilities.

**Table 3:** Themes and codes identified in pretest interviews.

**Theme**	**Theme definition**	**Code**	**Example**
Indication of a lack of motivation to revise	Comments that refer to specific contexts in which revision will not be attempted or continued	No revising if not required by teachers	“I don't have the habit of revising… I guess I'm just too lazy to do that. I don't even feel like to write an English paper, not to mention revising it. If the teacher does not require us to revise our writing, I wouldn't do that on my own initiative” “I believe that practice makes perfect, however, I don't think I can do that on my own. I need the teacher's comments and feedback to know what problems exist in my writing”
		No revising in the case of no authentic readers	“I rarely revise my paper… except when we were asked to rate each other's writing; otherwise, I felt less motivated to revise as nobody would read it” “I was even not sure whether the teacher would read it [the writing] or not. Why bother revising it?”
		Discontinuing revision when desirable scores are obtained	“It's important to meet the criteria stated in the writing marking rubric… I only revise my paper to narrow the gap between what is required and what I've achieved in the writing. That's the way to get a good mark in tests. I will stop revising, for example… the main points, if they have met the corresponding criteria, at least in my judgment, even if I know I have something better to say”
		Discontinuing revising when confronted with difficulties	“I guess I'm just not good at it (revision)… I often feel challenged when asked to revise ideas … so I just corrected errors in grammar and sentence structure. I don't think my paper improved much after I revised it” “It just seemed to be a lot of work…so I gave up doing it [revising] after tidying it up a little bit”
Indication of over-confidence in revision	Comments that imply an overestimation of one's ability to modify texts	Insisting on the use of words	“There is something I don't get when we're asked to read each other's work… they are very strict with the use of words. There was this time a classmate pointed out that I might use a word in a wrong way because he looked it up and didn't think the word had the meaning which I wanted to express. But, I kind of stick to my way of using it, I think it's right as long as it fits the context”
		Viewing revision as an easy task	“It's not that difficult to revise… I mean going through the words and sentences in paper and fix any errors, right? I'm good at that” “I would go over my writing every time I finished writing. It does not take long”

A cluster of responses is related to the unwillingness of participants to initiate or sustain efforts in revision in specific situations, thus forming the theme of indication of a lack of motivation to revise. Four codes are related to this theme: first, no attempts to revise on one's own initiative if teachers did not instruct them to revise; second, no attempt to revise on one's own initiative in absence of readers; third, discontinuing revision when desirable writing scores were obtained; and lastly, discontinuing revision when confronted with difficulties. The comments clustering around the first code, no attempts to revise if not being required by teachers, indicated that they were inclined not to engage in any forms of revision on their own initiative. As shown in the example in this code in Table 3, without the instruction of the teacher that specifically asked the student to revise, he was very likely to avoid it, suggesting a lack of intrinsic motivation in engaging in the act of revision itself. The phrase “too lazy to revise,” which appeared frequently in comments under this code, reflected their reluctance to engage in revision activities. One student claimed that he would not “push” himself to revise unless the teacher required them to do that; another student commented that he would not “bother” to revise if it was not an activity that was embedded in the writing course.

Students mentioned another external source for their motivation to revise, the presence of readers, forming the second code that related to students' reluctance to revise when readers were absent. As can be seen from the excerpt under this code in Table 3, the student would be more willing to revise if his writing would be read and evaluated by others such as peers. In cases where there were no authentic readers, the student was unlikely to initiate a revision. A frequently mentioned reason for this was that others could help to locate problems that one failed to notice, making revision “worth the time and efforts” as one student put it. It can be inferred that these students viewed revising on one's own as an activity that did not deserve much time and effort, indicating their lack of motivation.

Comments in the third code, discontinuing revision after desirable scores were obtained, indicated that student's motivation to revise was driven by better performance in writing tests. A typical comment, as presented in Table 3, indicates that the student pushes himself to revise to obtain a satisfactory mark on a writing test. The moment he realizes that he has met the criteria for reaching the score he wants, he will stop revising even if he has “something better to say,” implying that he possesses little intrinsic motivation for engaging in revision.

The final code in this theme related to these students' discontinuing their engagement with revision the instant that they met difficulties in the revising process. As can be seen from the example under the final code in Table 3, the student, who found it challenging to revise ideas in a text, turned to surface modification instead; it should be inferred from the statement that he might have attempted to improve the ideas in texts, but stopped further engagement in content revision the moment he ran into difficulties. The experience of failing to revise the content led him to conclude that he was “not good at it.”

Despite the indication of the unwillingness of interviewees to revise the content, evidence in support of their confidence in revising the surface features of texts was detected, which was encapsulated in the theme of indication of over-confidence in revision. Comments in the code of insisting on the use of words illustrated that these students might stick to their way of using words even if others pointed it out that they were used incorrectly. A typical response is included in Table 3 as an example; the student stated that he persisted in using the word even though his classmate “looked it up” and expressed his concern that it might not be used correctly. As evidenced by the fact that he “sticks to his way” instead of changing it as advised, the student might be overconfident in revising vocabulary in texts. Also, comments in the code of viewing revising surface features as an easy task, the other code in this theme, pointed to these students' overconfidence in their ability to revise at the local level of texts. A characteristic of the comments in this code is the use of phrases such as “I'm good at it…” or “it's not that difficult …,” as can be seen from the example in Table 3.

#### Post-test Interview

The interviewees indicated a greater willingness to revise the higher-level features of writing with the use of revision strategies and the procedures to carry them out. They also indicated the need for further practice in using the revision strategies to hone their revision skills, which contrasted with their overconfidence prior to the treatment. Table 4 describes the two themes that emerged in the posttest interviews with the codes subsumed under each: indication of willingness to revise higher-level features of texts and indication of efforts exerted and sustained in learning to revise.

**Table 4:** Themes and codes identified in post-test interviews.

**Theme**	**Theme definition**	**Code**	**Example**
Indication of willingness to revise higher-level features of texts	Comments that refer to students' interest or motivation to revise the higher-level features of texts, such as content and organization	Willingness to revise organization	“The whole point is to convince, to argue, so, I devote my attention to make it more logical, whether the ideas can be connected more logically” “When I went through my writing … read it through at the end, I realized that it didn't flow. I mean, the ideas were kind of … disconnected; I can't see the relationships between the ideas. That's what I should work on”
		Willingness to revise ideas	“It was not that easy to use at first…you have to carry it out step by step. But I have to say, it is very helpful after I get the hang of it; it helps me to modify the linking between sentences, to examine whether ideas are off the topic, things that I would never do if I haven't learnt the procedure. I'll use it more often; as they say, practice makes perfect. Then I can revise my writing really smoothly even in an exam” “…I wrote a paper and when I took a closer look at it, I realized that some sentences were off the topic and one of the main ideas was not relevant. So, I kind of rewrote a large part of it; I'm sure the revised paper is way better than the initial draft”
		Willingness to revise argument elements	“Revision has become more straightforward with the use of the criteria. I made very few changes before, I didn't really know what specific areas I should focus on except the errors in words or sentence structures. With the criteria, I know what to look for, like the reasons I used in the body part, sometimes they were not good enough. I pay extra attention to these areas and make more changes than I used to … they're helpful in identifying the areas I still need to work on”
Indication of efforts exerted and sustained in learning to revise	Comments that refer to initiate and sustain efforts to improve one's revision ability, such as using revision strategies or more practice	Initiating efforts in using the revision strategies	“I like the part where the teacher elaborated on how to use the strategy as well as how to select an appropriate tactic. It's really easy to follow, especially when the problem he was dealing with was one that I used to encounter. In that case, I thought about what I did during the previous revision experience and paid great attention to what he would do. I imitate the way he applied the revision tactic moving forward. That's why I believe practice could help to improve my revision ability” “They're really helpful … the steps, procedure, and everything. I'll definitely revise my writing because now I can see the point in doing that. I just did that this morning!”
		Sustaining efforts in practicing the use of the revision strategies	“I used to have no idea of how to revise my writing; I was completely… at a loss the first time when the teacher asked us to make changes to improve our paper (person). Now I feel like that… I think I have a much clearer idea of what I should do. I'm still trying the strategies, and I know I'm still not an expert in using them, but… I'm making progress, gradually” “I'll definitely use it [the scoring rubric] from now on; it provides a list of standards against which I can compare my text”

The first salient code in the theme of indication of willingness to revise higher-level features of texts is the willingness to revise across different levels of texts, reflecting students' emergent willingness to attend to global issues in texts rather than solely focusing on local issues. Students articulated their desire to improve the organization of ideas in texts. As can be seen from the example in Table 4, the student focused on whether the ideas in writing could be linked more logically. Equally, these students expressed their desire to improve the quality of ideas, as seen in comments categorized into the theme of willingness to revise higher-level features of texts, forming the code of willingness to revise ideas in texts. For example, one student stated that he modified ideas that were “off the topic” with the help of revision strategies as well as the procedures for carrying them out; he described idea revision as among the things he “would never do” before he learnt the strategies, and expressed being motivated to apply them in further practice, although he found them “not easy to use at first.”

Another cluster of comments pointed to the attention of students to argument elements in texts; students expressed a motivation to engage in extra efforts to revise argument elements in their texts in comments that were categorized into the code of willingness to revise argument elements. An example was provided under this code in Table 4; the student implied in his statement that he could revise with fewer efforts after the instruction as revision became “more straightforward,” especially about the evaluation and improvement of argument elements. He attributed this positive change to the use of genre-specific evaluation criteria, which helped him to “identify the areas” that needed modification when revising, such as “reasons in the body part”; this was in sharp contrast to how he used to focus solely on “errors in words or sentence structures.”

The second theme, an indication of efforts exerted and sustained in learning to revise, is subdivided into two areas: initiating efforts in practicing revision strategies and sustaining efforts in learning the use of revision strategies. The comments related to the first code expressed a view that the teacher's modeling of the revising process, particularly of how to select and apply the revision strategies, prompted these students' motivation to explore the use of these revision strategies. As can be seen from a typical response in this code in Table 4, the student in the example reported that the modeling made revising “easy to follow,” thus triggering his motivation to “imitate the way” the teacher used revision tactics when revising his writing. Similarly, another student also implied in his comments that the modeling provided an impetus for increasing his motivation to use the revision strategies. He stated that the instruction provided him with “more choices of what to change and how to do it,” implying that the instruction equipped him with a toolkit for revision, which helped him to revise more easily as he felt “more competent, maybe better at revision.”

These students also spoke of continuing engagement with the practice of revision strategies, forming the code of sustaining efforts in learning the use of revision strategies. As one student explained, although he knew that he had not fully mastered the use of the revision strategies, as evidenced by his comment that he was “not an expert in using them” (see Table 4), he believed that he was “making progress,” implying his willingness to continue using these strategies in further practice. Equally, these students articulated the need for further practice to improve their abilities to use the strategies. One participant used to believe that he “did a good job revising the paper” each time he finished writing. However, having learnt how to revise effectively, he realized that “there is a lot more to do,” which implied that he had formed more accurate judgments of his revision ability. A re-evaluation of his ability resulted in a conclusion that he still needed further practice, implying that he did not perceive his revision ability to be as high as he used to believe.

To sum up, compared with the pretest interviews, the posttest interviews showed that these students reportedly had become more efficacious in applying the revision strategies and expressed increased intrinsic interest in revision. Specifically, the students interviewed expressed an increased willingness to perform high-level revisions after the treatment, actions they used to find burdensome and thus avoided doing. They were also less likely to make negative statements, suggesting that they lacked motivation for revising.

## Discussion

The quantitative results obtained in this study showed a minimal variance in ratings of students for the self-efficacy scale after the SRSD instruction, indicating a limited instructional impact on the self-efficacy of students. These findings were at odds with previous studies, where positive effects of SRSD instruction on the writing self-efficacy of students were found (e.g., Teng and Zhang, 2020). A plausible explanation for such findings might be that students' psychological factors such as intrinsic motivational beliefs and self-efficacy, despite being dynamic, may require a long period to evolve and take effect (Dörnyei and Ushioda, 2011). In Teng and Zhang's (2020) study, their instruction lasted for 20 weeks; however, in the current study, the treatment group only received 8 weeks' instruction. Although the qualitative data showed the indicators of the SRSD-trained students' greater self-efficacy for text revision after instruction as they were less likely to make negative statements about their abilities to revise, it is possible that the interviewees might be relatively motivated to write as they were the first six students who volunteered to participate in the interviews. It is likely that students' self-efficacy for text revision might require more time to develop than was available in the current instructional program.

Nevertheless, previous studies in L1 writing have also reported no obvious relationship between SRL strategies and levels of self-efficacy (e.g., Graham et al., 1992; Graham et al.'s, 2005; Mills, 2012; MacArthur and Philippakos, 2013). A similarity shared by these previous studies is that the participants were found to not be able to accurately assess their capabilities, especially using a self-report self-efficacy scale, and thus causing mismatches between their performance and perceived abilities after instruction (e.g., Graham et al.'s, 2005). In Graham, Harris and Mason (2005) study, the participants' pretest self-efficacy judgments were unrealistically high; they did not demonstrate significant changes in their self-efficacy after instruction. Researchers have reported that students may judge themselves as capable of performing a task, but do not perform it; or they judge themselves as inefficacious, but then perform it. They described such students, whose judgments of what they were capable of doing bear little relation to their actual choice, as poorly calibrated (e.g., Pajares and Kranzler, 1995; Schunk and Pajares, 2002, 2010; Schunk and DiBenedetto, 2016). Students might possess poor calibration because they were not adequately aware of the task requirements and thus overestimated their capabilities to fulfill them (Wigfield et al., 2012). A substantial body of literature has revealed that writers, especially the unskilled, tend to have inaccurate judgments for assessing and predicting their capabilities (e.g., Klassen, 2002a,b).

Participants in the current study might also have overestimated their revision abilities, especially their capabilities to revise the high-level features of texts, at the outset. Their pretest ratings for the subscales were both a little bit over the median (see Table 2), although those high-level text revisions were more cognitively demanding than those at the lower level. There were also indicators of overconfidence in pretest interviews of students. These indicators of the overconfidence, however, disappeared in their posttest interviews; instead, they expressed a need for future practice to further improve their revision abilities. A possibility is that students formed more accurate judgments of their capabilities after participating in the SRSD program, which helped them to learn task requirements and gain task familiarity. It is likely that they overrated themselves before instruction and, with the relatively more accurate estimation of their abilities in the posttest, the over-time comparison of their responses to the L2TRSS did not reveal any statistically significant changes. The instructional procedures designed to improve the self-efficacy of students, such as modeling, scaffolding, and reinforcing learners' efforts, were included in the design of the current SRSD-based revision instruction. During the instructional process, the teachers modeled the revision process and demonstrated explicitly how to use revision and self-regulation strategies. This kind of scaffolding could help to build confidence in students as it would ease their cognitive burden and make the learning tasks more manageable. The importance of student effort in learning was emphasized and rewarded throughout the instructional program to facilitate the development of positive attitudes toward revision. Hence, the results obtained in this study, along with those reported in previous SRSD studies (e.g., MacArthur et al., 2015), pointed to the positive effects of SRSD on the self-efficacy of students. For those students who tended to lack confidence and struggle with motivation, SRSD has shown to raise their self-efficacy (e.g., MacArthur et al., 2015; see also Harris and Leeming, 2021); however for those students who were likely to overestimate their abilities (e.g., García-Sanchez and Fidalgo-Redondo, 2006), SRSD helped them to form more accurate judgments about their capabilities.

## Conclusion

This study attempted to explore the effects of the SRSD model on self-efficacy of college students in the domain of text revision in an EFL context. Although conclusive evidence supporting students' enhanced self-efficacy for text revision after the SRSD revision instruction was not obtained, our results suggest that SRSD strategy employment might have helped EFL students to form more accurate judgments about their capabilities to revise the following instruction. An implication for educational practice in teaching revision is that EFL instructors are advised to be aware of learners' overconfidence, particularly about difficult tasks such as text revision, when feedback suggesting poor performance has been provided. Moreover, it is recommended to monitor self-efficacy judgments of students along with their performance in case either unrealistically high levels of self-efficacy or undesirably low levels of efficacy negatively influence the learning behavior of students. Researchers (e.g., Schunk and DiBenedetto, 2016) posited that the incongruence between self-efficacy and actual performance could arise when learners lack task familiarity and have a limited understanding of what capable performance entails. In such cases, a high level of self-efficacy could be problematic as learners feel unrealistically overconfident and are reluctant to allocate needed efforts and resources since they believe this is not necessary (MacArthur, 2015; Schunk and DiBenedetto, 2016). As the SRSD instruction could enable students to form relatively accurate judgments about their revision abilities, it could be applied to tackle the problem of overconfidence in the domain of text revision at the tertiary level.

Several limitations of this study should be borne in mind when interpreting the findings. First, convenience sampling was used to recruit participants in the current study for easy accessibility, and hence, the two groups were of different majors, which might have functioned as a variable influencing their self-efficacy level. Although the two groups reported similar levels of self-efficacy at the outset, they developed differently in self-efficacy levels because of their various majors. Another limitation, as discussed above, is that the interviewees might not be adequately representative of the population. For ethical considerations, to ensure that every student had an equal opportunity to participate, the first six students who volunteered were selected, regardless of their writing proficiency or self-efficacy level. These volunteers might be relatively motivated to write. Nevertheless, in line with the primary objective of the study, namely, the possible changes in the indicators of self-efficacy, the analysis and interpretation of interview data focused on the variance in the self-efficacy. Also, previous studies have shown that it is the students of relatively low writing proficiency and weak motivation that benefitted the most from the SRSD instruction (De La Paz and Sherman, 2013). In this study, positive changes were found in students interviewed, who were assumed to be of relatively strong motivation; it is likely that students who were of weaker motivation than the interviewees might demonstrate similar, positive changes. Because of these limitations, the findings of the current study might not be generalized to other SRSD studies where significant instructional effects were reported. Further research should include interviewees of various self-efficacy levels and explore whether their self-efficacy would change to different degrees resulting from the SRSD model.

Besides addressing the limitations of the current study, further research is required to explore several issues. First, further studies should extend the duration of the SRSD instruction to allow the self-efficacy of students to fully develop. Second, to understand whether a lack of task familiarity accounts for overestimates, future studies could explore whether improvements in students' task knowledge are related to changes in their self-efficacy. A better understanding of the reasons explaining such overestimation could shed lights on how to counter the mismatch between students' actual performance and perceived capabilities. Also, other motivational factors need to be examined in future SRSD studies to better understand the instructional effects of the SRSD model as well as to understand the complex operation of self-efficacy. Prior studies have explored other motivational factors such as mastery motivation, and positive results were found in both self-efficacy and mastery motivation (MacArthur et al., 2015).

## Data Availability Statement

The original contributions presented in the study are included in the article/Supplementary Material, further inquiries can be directed to the corresponding author/s.

## Ethics Statement

The studies involving human participants were reviewed and approved by The University of Auckland Human Participants Ethics Committee. The patients/participants provided their written informed consent to participate in this study.

## Author Contributions

JC and LZ conceived and designed the study. JC collected the data and drafted the manuscript. LZ finalized the manuscript for submissions. All the authors revised the manuscript.

## Conflict of Interest

The authors declare that the research was conducted in the absence of any commercial or financial relationships that could be construed as a potential conflict of interest.
