# Corrigendum: Impacts of Self-Regulated Strategy Development-Based Revision Instruction on English-as-a-Foreign-Language Students' Self-Efficacy for Text Revision: A Mixed-Methods Study

**DOI:** 10.3389/fpsyg.2021.747252

**Published:** 2021-08-18

**Authors:** Jing Chen, Lawrence Jun Zhang, Xiao Wang, Tingting Zhang

**Affiliations:** ^1^College of Foreign Languages, Huazhong Agricultural University, Wuhan, China; ^2^Faculty of Education and Social Work, University of Auckland, Auckland, New Zealand; ^3^Teaching and Learning Group, The Royal New Zealand Police College, Wellington, New Zealand

**Keywords:** self-regulated strategy development, self-efficacy, text revision, EFL writing, classroom intervention

In the published article, there was an error in affiliation 1 as published. Instead of “School of Foreign Languages, Central China Agricultural University, Wuhan, China”, it should be “College of Foreign Languages, Huazhong Agricultural University, Wuhan, China”.

The authors apologize for this error and state that this does not change the scientific conclusions of the article in any way. The original article has been updated.

## Publisher's Note

All claims expressed in this article are solely those of the authors and do not necessarily represent those of their affiliated organizations, or those of the publisher, the editors and the reviewers. Any product that may be evaluated in this article, or claim that may be made by its manufacturer, is not guaranteed or endorsed by the publisher.

