# Supplementary material for: Impacts of Self-Regulated Strategy Development-Based Revision Instruction on English-as-a-Foreign-Language Students' Self-Efficacy for Text Revision: A Mixed-Methods Study
Source: Front Psychol. 2021 Jul 15;12:670100. doi: 10.3389/fpsyg.2021.670100 (PMC8321093; doi:10.3389/fpsyg.2021.670100)
Supplement: Supplementary file 1 [file Data_Sheet_1.pdf]

Appendix A: The Second Language Text Revision Self-Efficacy Scale (L2TRSS)

Using the scale shown below, please rate how certain you are that you can do each of the things described below by choosing an appropriate number ranging from 0 to 10.

0	1	2	3	4	5	6	7	8	9	10
I cannot do it					Moderately certain that I can do					Highly certain that I can do

Confidence score (0-10)

e.g., I can write a simple sentence with good grammar. 7

Self-efficacy for low-level text revision

1. I can correct misspelled words when revising my essay. __________
2. I can revise punctuation marks in my essay to make their use more accurate. __________
3. I can correct grammatically incorrect words in my essay. __________
4. I can correct wrong parts of speech (e.g., nouns, verbs, adjectives, etc.) in my essay. __________
5. I can revise sentences in my essay to ensure they are grammatically correct. __________
6. I can revise cohesiveness or connection among sentences (e.g., linking words such as however, on the other hand) in my essay to make the ideas flow more fluently. __________

Self-efficacy for high-level text revision

7. I can re-arrange the content in my essay to organise ideas better into paragraphs (e.g., each paragraph deals with one overall idea). __________
8. I can re-organise ideas in each paragraph to make sure they are presented in a more logical way. __________
9. I can revise language features (e.g., tense, tone, and vocabulary) in my essay to make them more appropriate to the audience (e.g., to consider the topic seriously, and to use respectful tone and formal language). __________
10. I can revise the language in my essay to make the arguments more objective (e.g., to use passive structures). __________
11. I can revise the language in my essay to make it more persuasive to the audience (e.g., to include emotive vocabulary such as “imagine that” or to refer to the reader “you would”). __________
12. I can revise my essay to ensure it contains all the core argument elements (e.g., position, main points, evidence, re-statement). __________
13. I can rewrite to state my position more clearly. __________
14. I can add more elaboration of main points when revising my essay (e.g., elaboration includes examples based on evidence, experience, or both). __________
15. I can delete examples or evidence that are irrelevant to the argument and add relevant ones when revising my essay. __________
16. I can delete content in my essay that is not closely related to the topic. __________
17. I can delete information in my essay that contributes little to the argument. __________
