# Supplementary material for: Impacts of Self-Regulated Strategy Development-Based Revision Instruction on English-as-a-Foreign-Language Students' Self-Efficacy for Text Revision: A Mixed-Methods Study
Source: Front Psychol. 2021 Jul 15;12:670100. doi: 10.3389/fpsyg.2021.670100 (PMC8321093; doi:10.3389/fpsyg.2021.670100)
Supplement: Supplementary file 2 [file Data_Sheet_2.pdf]

Appendix B: Pre- and post-test semi-structured interview schemes

Pre-test

Topic one: Previous revision experience

1. Do you revise your writing?
   - If yes, could you describe how you used to revise your writing?
   - If no, why?
2. Do you think revising is important? Why or why not?
3. What do you think a good reviser will do during the revising process?
4. Have you heard of revision strategies?
   - If yes, what are the revision strategies you know?
   - And do you know how and when to use them? If yes, please explain.

Topic two: self-efficacy for text revision

1. Do you think you are good at revising?
   Why or why not?
2. Do you feel motivated to revise your writing?
   Why or why not?
3. Do you think you can improve your revision skills if you practice more in the future?
   Why or why not?

Post-test

Topic one: current understanding of revision and one's approach to revising

1. What do you think effective revision involves?
2. Do you use revision strategies?
   -If yes, what are they?
   -And are there any revision strategies you did not use prior to the treatment? If yes, please specify the different revision strategies you use now.
3. Could you describe how and when to use revision strategies?
4. What do you think a good reviser does in the revising process?
5. Has your understanding of what a good reviser does in the revising process changed after the treatment? If yes, what are the changes in your understanding?

Topic two: self-efficacy for revision

1. Do you think you've become more competent in revision after the treatment?
   -Why or why not?
2. Do you feel more motivated to revise after the treatment?
   -If yes, what makes you think this?
3. Do you think you can improve your revision skills if you practice more in the future?
   -Why or why not?
